# Long-term mindfulness meditation increases occurrence of sensory and attention brain states

**DOI:** 10.3389/fnhum.2024.1482353

**Published:** 2025-01-06

**Authors:** Daniel Yochai Panitz, Avi Mendelsohn, Joana Cabral, Aviva Berkovich-Ohana

**Affiliations:** ^1^Sagol Department of Neurobiology, Faculty of Natural Sciences, University of Haifa, Haifa, Israel; ^2^The Institute of Information Processing and Decision Making (IIPDM), University of Haifa, Haifa, Israel; ^3^Integrated Brain and Behavior Research Center, University of Haifa, Haifa, Israel; ^4^Life and Health Sciences Research Institute (ICVS), School of Medicine, University of Minho, Braga, Portugal; ^5^Centre for Eudaimonia and Human Flourishing, Linacre College, University of Oxford, Oxford, United Kingdom; ^6^ICVS/3B’s - Portuguese Government Associate Laboratory, Guimarães, Portugal; ^7^School of Therapy, Counseling and Human Development, Faculty of Education, University of Haifa, Haifa, Israel; ^8^Edmond Safra Brain Research Center, Faculty of Education, University of Haifa, Haifa, Israel

**Keywords:** dynamic functional connectivity, functional MRI, mindfulness meditation, brain states, resting state networks, LEiDA

## Abstract

Interest has been growing in the use of mindfulness meditation (MM) as a therapeutic practice, as accumulating evidence highlights its potential to effectively address a range of mental conditions. While many fMRI studies focused on neural activation and functional connectivity during meditation, the impact of long-term MM practice on spontaneous brain activity, and on the expression of resting state networks over time, remains unclear. Here, intrinsic functional network dynamics were compared between experienced meditators and meditation-naïve participants during rest. Our analysis revealed that meditators tend to spend more time in two brain states that involve synchrony among cortical regions associated with sensory perception. Conversely, a brain state involving frontal areas associated with higher cognitive functions was detected less frequently in experienced meditators. These findings suggest that, by shifting attention toward enhanced sensory and embodied processing, MM effectively modulates the expression of functional network states at rest. These results support the suggested lasting effect of long-term MM on the modulation of resting-state networks, reinforcing its therapeutic potential for disorders characterized by imbalanced network dynamics. Moreover, this study reinforces the utility of analytic approaches from dynamical systems theory to extend current knowledge regarding brain activity and evaluate its response to interventions.

## Introduction

Mindfulness meditation (MM) is a practice of momentary presence, without interpretation or judgment (Jacobson et al., 1995). It draws its roots from eastern practices and philosophies, especially Theravada Buddhism (Olendzki, 2010). Mindfulness-based interventions (MBIs) have been found to improve a wide range of cognitive functions, including emotion regulation (Kral et al., 2018; Zhang et al., 2019), attentional control (Prakash, 2021; Sumantry and Stewart, 2021), sleep quality (Rusch et al., 2019), and wellbeing (Querstret et al., 2020; Zhang et al., 2021; Zollars et al., 2019). Implementation of MBIs have also shown promising results in alleviating various mental conditions, including anxiety (Crowley et al., 2022; Zhihong et al., 2017), depression (Parmentier et al., 2019; Reangsing et al., 2021), PTSD (Haider et al., 2021; Sun et al., 2021), ADHD (Gu et al., 2021; Geurts et al., 2021), and schizophrenia (Chai et al., 2022; Özdemir and Budak, 2022).

In the past decades, the mechanisms underlying the benefits of MM practice were at the center of a plethora of behavioral and neuroimaging studies. Particularly, it has been suggested that MM includes three components that interact closely to constitute a process of enhanced self-regulation: enhanced attentional control, improved emotion regulation, and altered self-awareness (diminished self-referential processing and enhanced body awareness; Hölzel et al., 2011; Tang et al., 2015). On the neural level, there is growing evidence that MM is related with the interplay between various cortical networks (see reviews Fox et al., 2016; Sezer et al., 2022). Particularly, there is emerging evidence for the impact of MM on the connectivity between nodes of different functional networks (Sezer et al., 2022). These include increased functional connectivity (FC) between the posterior cingulate cortex (PCC) as a node of the default mode network (DMN), and the dorsolateral prefrontal cortex (dlPFC) as a node of the frontoparietal network (FPN), suggested to affect attention control, as well as decreased connectivity between the cuneus and nodes of the salience network, which are relevant to self-awareness.

Traditionally, resting-state fMRI studies evaluate the level of functional connectivity (FC) between two brain areas by the correlation between their signals over the entire recording time. However, a growing number of studies has demonstrated that functional connections are not static, switching between periods of low and high correlated activity, during which a functional network is assumed to be active (Calhoun et al., 2014; Chang and Glover, 2010; Hutchison et al., 2013; Kringelbach and Deco, 2020; Preti et al., 2017; Zalesky et al., 2014). Despite remaining active only for short periods of time, functional networks reoccur repeatedly over time and across subjects during rest (Vohryzek et al., 2020). Notably, the proportion of time a given functional network activates during a scan has been shown to better discriminate between different psychiatric, pharmacological and behavioral conditions than simple static FC measures, indicating that the brief but consistent recurrence of these network patterns has a close relationship with brain function. Being a relatively recent development, only a few studies have explored meditation-related changes in cortical network dynamics, and the evidence is only starting to emerge, both on the state (short-term, during meditation) and trait (long-term, during non-meditative conditions) levels. Some studies focused on dispositional mindfulness, a personality trait referring to the innate capacity of paying and maintaining attention to present-moment experiences with an open and nonjudgmental attitude (Brown and Ryan, 2003). Particularly, two studies have linked dispositional mindfulness with more frequent transitions between brain states (Lim et al., 2018; Marusak et al., 2018), and another has shown that compared to non-practitioners, the dynamical regime of the brains of experienced meditators exhibit higher metastability during rest (Escrichs et al., 2019). Two additional studies pointed to the potential of MM interventions to alter the dynamics of internetwork connectivity, namely between the salience network, the FPN (equivalent to the executive network), and the DMN (Bremer et al., 2022; Mooneyham et al., 2017). A case study has suggested that MM has the potential to reconfigure functional network architecture, revealing differences in community affiliation of various brain regions from the FPN to other networks, primarily to the DMN (Kajimura et al., 2020). Finally, another study has associated higher dispositional mindfulness with attenuated response to a social stressor, accompanied by a smaller decrease in a connectivity state that is characterized by strong connectivity within the DMN and anti-correlations between areas of this network and areas of other networks (Teng et al., 2022). This connectivity state resembles a state found in a previous study, in which participants with lower dispositional mindfulness spent significantly less time compared to participants with high dispositional mindfulness (Lim et al., 2018).

In this work, we explore the effects of long-term MM on spontaneous brain activity by comparing the probability of occurrence of intrinsic networks on previously acquired resting-state fMRI scans of long-term meditators and age-matched control participants (Berkovich-Ohana et al., 2016a). While similar to the work of Escrichs et al. (2019), which examined the metastability of functional connectivity dynamics between meditators and non-meditators, the current study focuses on differences between these populations in the occurrence of individual brain states. Additionally, we explore potential relationships between the measured neural signatures with age and meditation expertise. This builds on previous studies that have shown that neural profiles of practitioners change differently as they age (Luders, 2014) and the amount of training experienced throughout the years (e.g., Berkovich-Ohana et al., 2020). Finally, we also examined the correlations between the occurrence of brain states and the Mysticism Scale (Hood, 1975). This scale assesses various experiential dimensions, including sense of unity, transcendence of time and space, ineffability, and feelings of sacredness, often associated with meditation practices (Taves, 2020). Hood’s Mysticism scale is also a measure of ego-dissolution, an alteration to the sense of self experienced during deep meditative states (Millière et al., 2018), hence it was used here to estimate changes in self-awareness.

The occurrence of intrinsic networks is quantified in this study using Leading Eigenvector Dynamics Analysis (LEiDA), an algorithm that captures patterns of FC over time in fMRI data based on instantaneous phase relationships between brain regions (Cabral et al., 2017; Vohryzek et al., 2020; Deco et al., 2019; Lord et al., 2019). These patterns of phase relationships detected at each time point are clustered into a repertoire of states, revealing that brain activity at rest exhibits a robust repertoire of patterns that recur over time and across participants, spatially overlapping with previously defined resting-state networks. A key measure that can be calculated by this procedure is the probability of a given state occurring during the scan, reflecting the fraction of the time participants spent in each state throughout the entire resting state scan. This measure is statistically compared between groups, illuminating the differences that may be induced by long-term MM practice.

## Materials and methods

### Participants

Twenty-one long term practitioners of MM and 23 meditation-naïve control participants underwent fMRI scanning as part of a previous study (Berkovich-Ohana et al., 2016a), including several participants from a pilot study that was run with the same settings. All participants were Caucasian, right-handed, having University level education, and with no history of neurological or psychiatric disorders. Four long term meditators and four control participants were excluded from the current study due to missing data, leaving 17 meditators (MM, mean ± SD age 41.9 ± 9.3 y.o., 6 females; see Table 1) and 19 control participants (C, mean ± SD age 40.3 ± 10.98 y.o., 7 females; see Table 1). We have considered the size of our sample as sufficiently reliable, based on previous studies, which showed that for a liberal threshold of 0.05, about 12 subjects were required to achieve 80% power at the single voxel level for typical activations (Desmond and Glover, 2002; see also Friston, 2013 for a critical view on large sample sizes and over-powered studies). In addition, we also calculated the effect size for each instance of significant between-groups effect (explained further on).

**Table 1:** Demographic information.

	Meditators (MM)	Controls (C)
Number of participants	17 (6 females)	19 (7 females)
Age in years (mean ± SD)	41.9 ± 9.3	40.3 ± 10.98
Meditation experience in years (mean ± SD)	15.2 ± 5.22	NA
Meditation experience in hours (mean ± SD)	8,442 ± 8430.42	NA
Meditation experience range in hours (minimum-maximum)	936–29,293	NA

SD, standard deviation; NA, not applicable.

The MM group consisted of long-term practitioners (mean ± SD 8442 ± 8430.42 h of formal practice, range 936–29, 293 h of formal practice over mean ± SD 15.2 ± 5.22 years; see Table 1). Hours of formal practice calculations included both accumulated hours in formal retreats, as well as accumulated daily practice throughout the years. All the meditators practice mindfulness meditation according to the Satipatthana and Theravada Vipassana traditions and were mostly recruited via the Israeli Insight Society TOVANA. All participants provided written informed consent for their participation. The experimental procedures were approved by the Tel Aviv Sourasky Medical Center - Ichilov Hospital ethics committee. This study was aligned with the Declaration of Helsinki and performed in accordance with the relevant guidelines.

### Experimental design

Before entering the scanner, participants completed Hood’s Mysticism Scale (Hood, 1975). MRI images were acquired on a 3 T Trio Magnetom Siemens scanner, at the Weizmann Institute of Science, Rehovot, Israel. Participants underwent a T1-weighted 3D Magnetization Prepared - RApid Gradient Echo (MP-RAGE) sequence, which was used for anatomical segmentation and template normalization (1x1x1mm3 resolution, TR = 2,300 ms, TE = 2.98 ms, TI = 900 ms, and FA = 9°), followed by resting-state fMRI (T2*-weighted, obtained with gradient echo planar imaging sequence), which was acquired continuously for 9 min (TR = 3,000 ms, TE = 30 ms, FA = 90°, inplane resolution—3x3mm^2^, slice thickness—3 mm, 46 axial slices; see also Berkovich-Ohana et al., 2016b; Berkovich-Ohana et al., 2016a). This scanning sequence is sensitive, but not exclusively, to the BOLD signal response (Chen et al., 2020). The participant’s head was placed on a foam cushion for stabilization, and MR compatible earphones (MR confon, Magdeburg, Germany) that substantially reduce external noise were placed on the ears. Participants were instructed to rest with their eyes closed, without falling asleep. In order to rule out cases of sleep or meditation practice during the resting-state, each participant was interviewed while in the scanner immediately after the conclusion of the scanning session via an intercom, using a semi structured interview. The participants were asked to describe their experience and thought content—a procedure which lasted around 5 min (Berkovich-Ohana et al., 2016a). In addition, each participant performed a visual recognition memory task (Hasson et al., 2003), the results of which have been previously published (Berkovich-Ohana et al., 2016b; Berkovich-Ohana et al., 2016a).

### fMRI data preprocessing

The anatomical MRI and resting state fMRI scans of the participants were acquired in DICOM format and converted into NIFTI files in the BIDS format using dicm2nii conversion tools^1^ in MATLAB. The fMRI data in this manuscript were preprocessed using *fMRIPrep* 20.2.1 with default settings (Esteban et al., 2019; Esteban et al., 2022a), which is based on *Nipype* 1.5.1 (Gorgolewski et al., 2011; Esteban et al., 2022b). Anatomical data preprocessing included the following steps: correction for intensity non-uniformity, skull stripping, brain tissue segmentation, brain surface reconstruction, and normalization to standard Montreal Neurological Institute (MNI) space. Preprocessing of the resting state fMRI T2* scans included correction of head motion, slice-time correction, co-registration to structural image and non-linear spatial normalization to MNI space. Many internal operations of *fMRIPrep* use *Nilearn* 0.6 (Abraham et al., 2014), mostly within the fMRI processing workflow (see the Supplementary methods section in the Supplementary materials for additional details).

The data used here was only minimally preprocessed, without special care to remove non-neuronal components, and therefore no assumptions are made regarding the origins of the fluctuations in the fMRI signal (Chen et al., 2020). The aim of the current study is to examine potential differences in functional connectivity dynamics between veteran practitioners of mindfulness meditation and meditation-naïve individuals, without separating between neuronal and non-neuronal sources.

### Cortical parcellation

The fMRI signals obtained at the voxel level were parcellated into *N* = 360 cortical parcels using the Cole-Anticevic Brain Network Parcellation (CAB-NP), allowing this work to benefit from the higher precision of the multimodal Glasser parcellation scheme (Glasser et al., 2016), while also associating each parcel with an established resting-state functional network (Ji et al., 2019). Since our data lacks the T2-weighted image required for the Human Connectome Project (HCP) pipelines, the relevant images were first converted from T1-weighted space into CIFTI format using Ciftify toolbox version 2.0.3 (Dickie et al., 2019), which uses a surface-based pipeline similar to that of the HCP (Glasser et al., 2013). The resulting parcellated data were stored N×T matrices in MATLAB-compatible files for the next stages of the analysis, where N=360 is the number of cortical parcels and T=180 is the number of volumes in each scan.

### Leading eigenvector dynamics analysis

To detect recurrent states of functional connectivity (FC), first the mean time-series of each of the N=360 parcels was transformed into an analytic signal (represented, at each instant of time, by a phase $\theta \left(n,t\right)$ and an amplitude $A\left(n,t\right)$ , where $n\in \left\{1,\ldots ,360\right\}$ and $t\in \left\{2,\ldots ,T-1\right\}$ ) using the Hilbert transform. The first and last volumes were excluded due to possible signal distortions induced by the Hilbert transform (Vohryzek et al., 2020). The dynamic phase coherence (*dPC*) was computed by estimating the phase alignment between pairs of brain regions *n* and *p* at each timepoint *t*, using the cosine similarity equation:


$$dPC\left(n,p,t\right)=\cos\left(\theta \left(n,t\right)-\theta \left(p,t\right)\right)$$

The dynamic phase coherence *dPC* is a tridimensional tensor with size $N\times N\times T^{\prime }$ obtained for each scan, where N is the number of brain parcels and $T^{\prime }=178$ is the number of time points in each fMRI scan. The *dPC* values range between −1 and 1, where 1 corresponds to the cases where the signals in brain regions n and p , at timepoint t have the same phase, $dPC\left(n,p,t\right)=\cos\left(0^{{}^{\circ}}\right)=1$ , whereas −1 corresponds to a phase difference of half a cycle (or 180°).

The phase relationships at each instant of time can be captured with reduced dimensionality and increased sensitivity using the Leading Eigenvector Dynamics Analysis (LEiDA) algorithm (Cabral et al., 2017). LEiDA consists of extracting the leading eigenvector, $V_{1}\left(n\right)$ , of the *dPC* matrices at each timepoint *t*. The leading eigenvector $V_{1}$ is a vector with size Nx1 that captures the dominant pattern of phase relationships between brain regions. Each of the *N* elements of the vector $V_{1}\left(n\right)$ represents how the signal in each brain region *n* is aligned in phase with respect to the other brain regions. Elements with the same sign (positive or negative) in the leading eigenvector indicate regions that are phase-aligned and can be considered part of the same community. The magnitude of the elements $V_{1}\left(n\right)$ indicates the extent to which the respective brain region *n* belongs to its community. Given that the relative sign in eigenvectors is arbitrary, a convention is applied such that the largest community has a negative sign, while the smallest community has a positive sign (Eraifej et al., 2023; Farinha et al., 2022; Hancock et al., 2022).

### Detection of phase-locking states in fMRI signals

Recurrent states of phase coherence were detected by applying a *k-*means clustering algorithm on all 6,408 leading eigenvectors obtained from the *dPC* matrices of all 36 participants. The *k*-means algorithm serves to detect recurrent patterns in the data, but requires determining *a priori* the number of clusters *k*. In the resting state literature, the number of functional networks is unclear, and the current consensus is that there is some hierarchy, which means that increasing the level of granularity might reveal more specific functional networks. The aim of this work is to attempt the detection of connectivity patterns, the occurrence of which differs between meditators and controls. To perform such an exploratory analysis, the k-means algorithm was run with *k* ranging from 2 to 20 and using the cosine distance as a measure of similarity between eigenvectors. Focusing on connectivity patterns that are not exclusive to a specific partitioning model, we opted not to rely on the metrics that are usually used to determine the optimal k, such as the Elbow method or Silhouette coefficient. For each value of *k*, this algorithm partitions the eigenvectors into *k* clusters. Averaging all the eigenvectors assigned to a given cluster returns the cluster centroid, $V_{c}$ , with size *Nx1*, which is considered to represent a state of phase-locking, or PL state, in brain signals. This term is distinct from the phase locking value (PLV), which is used in other studies (e.g., Ponce-Alvarez et al., 2015). In essence, the centroids of the various PL states capture modes of phase synchronization across brain regions, where elements with the same sign represent in-phase locking, and anti-phase locking is represented by elements with opposite signs (Hancock et al., 2022). It has been consistently found that the smallest subset of regions with the same sign in these cluster centroids exhibit a significant spatial overlap with known intrinsic connectivity networks and therefore this smaller subset of brain regions is considered as representative of the functional subsystems activated in each PL state (Eraifej et al., 2023; Larabi et al., 2020; Lord et al., 2019).

### Probability of occurrence and between-groups differences

The clustering assigns to each timepoint a single PL state, making it possible to characterize each fMRI scan by its sequence of states over time. Using the state time course, we calculated the probability of occurrence of each state, which is simply the number of timepoints assigned to a given PL state divided by the total number of timepoints in each scanning session. For each partition model (i.e., for different numbers of clusters *k*) the probabilities of each PL state were calculated for each subject.

Differences in probabilities of occurrence between the groups were statistically assessed using a permutation-based non-parametric two sample hypothesis test, which estimates the null distribution by permutations of group labels, instead of relying on the test-type standard distributions. For each of 10,000 permutations, a t-test was applied to compare populations, and a *p*-value is returned. Correction for multiple comparisons was performed for each partitioning model separately using the conservative Bonferroni correction, i.e., by dividing the threshold for significance by the number of states in the model.

As an additional means of validating the robustness of our results, the effect size of each significant between-groups difference was estimated (Farinha et al., 2022). This was done using the biased form of Hedges’ g statistic, also known as Cohen’s d, which measures the effect size for differences between means (Hedges, 1981), and calculated as


$$g=\frac{{\bar{\underline{x}}}_{1}-{\bar{\underline{x}}}_{2}}{s^{*}}$$

where $s^{*}$ is the pooled standard deviation, which is computed as


$$s^{*}=\sqrt{\frac{\left(n_{1}-1\right)s_{1}^{2}+\left(n_{2}-1\right)s_{2}^{2}}{n_{1}+n_{2}-2}}\text{.}$$

Having found PL states that exhibit significant between-groups differences in terms of their probability of occurrence in their respective partitioning models, these PL states were grouped into coupling modes (CMs). Each CM represents a recurring mode of cortical phase synchronization that can be found in several partitioning models, and therefore can be considered independent of the granularity of the model. This grouping was performed on the basis of visual inspection and validated by computing correlations for each pair of relevant PL states between their respective centroids, which stand for the connectivity pattern they represent.

### Correlating probabilities of occurrence with age, meditation expertise, and Hood’s mysticism scale

A core mechanism of mindfulness meditation is changes to self-awareness (Tang et al., 2015), mainly studied via alterations to the DMN and largely thought to support the sense of self (Northoff et al., 2006). Importantly, the same cohorts reported in this study were previously shown to exhibit significant differences in various nodes of the DMN, both on the functional level (Berkovich-Ohana et al., 2016a), as well as the structural level (Berkovich-Ohana et al., 2020). In order to assess any potential link between functional connectivity dynamics and changes in self-awareness, all participants answered Hood’s Mysticism Scale (Hood, 1975), which is considered to be a measure of ego-dissolution, which is an alteration to the sense of self experienced during deep meditative states (Millière et al., 2018). This questionnaire is composed of eight subscales that relate to different qualities of mystical experiences: Ego quality refers to experiencing the loss of the sense of self while maintaining consciousness (e.g., “I have had an experience in which something greater than myself seemed to absorb me”); Unifying quality refers to the perception of unity among separated objects (e.g., “I have had an experience in which I realized the oneness of myself with all things”); Inner Subjective quality refers to perceiving an inner subjectivity even to material objects (e.g., “I have had an experience in which all things seemed to be conscious”); Temporal/Spatial quality refers to the modification of the temporal and spatial parameters of the experience (e.g., “I have had an experience which was both timeless and spaceless”); Noetic quality refers to treating the experience itself as a valid source of information (e.g., “I have had an experience in which a new view of reality was revealed to me”); Ineffability refers to the inability to verbally express the nature of the experience (e.g., “I have had an experience that cannot be expressed in words”); Positive Affect refers to the experience of joy or blissful happiness (e.g., “I have experienced profound joy”); and Religious quality, which refers to the sacredness of the experience (e.g., “I have had an experience which I knew to be sacred”). Correlations were calculated between the probabilities of occurrence for each PL state and participants’ scores on the various subscales of Hood’s Mysticism Scale, along with the mean score across the different subscales, both for the whole sample and for each group separately.

Considering that expertise in meditation develops across prolonged timescales, spanning years of practice and training, correlations were also calculated between the probabilities of occurrence for the different PL states and participants’ assessed meditation expertise (by years and by hours) only within the MM group. Important factors that may contribute to, or confound, findings of interest, relate to the age of the participants (age-related changes in the neural profiles of practitioners have been shown to differ from those among meditation-naive individuals; Luders, 2014), and the amount of time of training that they experienced throughout the years (e.g., Berkovich-Ohana et al., 2020). Therefore, the probabilities of occurrence of the various PL states were also correlated with participants’ age, aiming to separate between changes in functional connectivity dynamics that relate to age from those that relate to the accumulation of experience in the practice of meditation. Similarly to the correlations with the scores of Hood’s Mysticism Scale, the correlations between the probabilities of occurrence and age were computed both for the whole sample and for each group separately, while the correlations with meditation experience were computed only for the meditators group. All correlations were corrected for multiple comparisons for each partitioning model separately, using Holm-Bonferroni corrected *p*-values.^2^

## Results

### Post-scan interviews

The participants were explicitly requested not to use meditation techniques during the resting-state scans. In order to rule out the possibility that they did, as well as to collect incidents of drowsiness, all participants were interviewed immediately after the scan about their experience during it (Berkovich-Ohana et al., 2016a). None of the participants reported drowsiness, and more importantly, none of the MM practitioners reported entering a meditative state, such as anchoring on the breath or body sensations, which are the typical instructions in MM practice (Gunaratana, 2002).

### States of FC detected across participants

**Figure 1:**
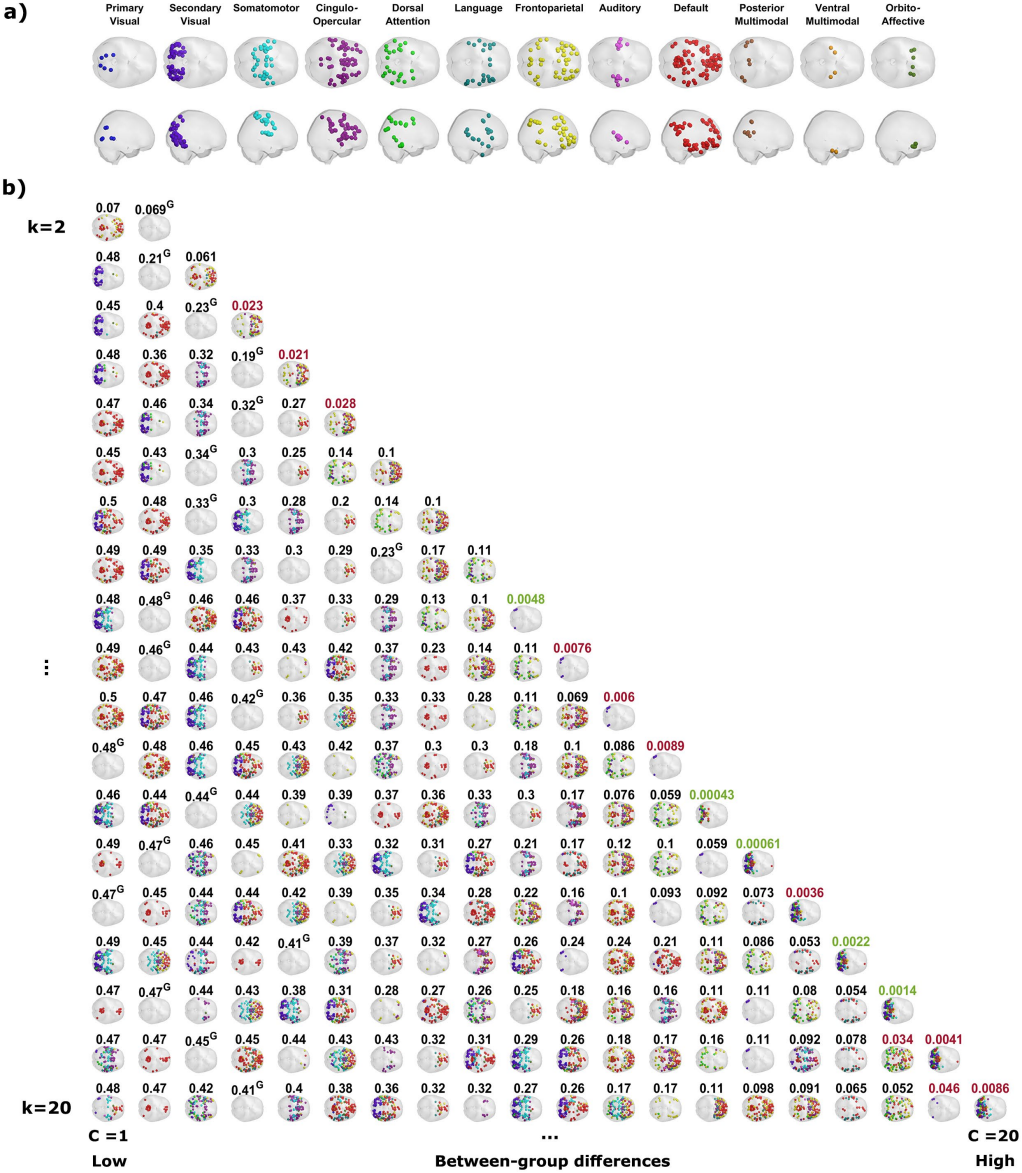
Recurring functional connectivity patterns detected by partitions into a varying number of states. **(A)** The network affiliation of each brain parcel according to Ji et al. (2019). **(B)** The clustering procedure reveals the phase-locking (PL) states for each partition model, plotted as separate rows. The PL states are represented as a set of spheres at the center of gravity only of the cortical parcels that are in anti-phase with respect to the rest of the brain. Each sphere is colored to match the established resting state network to which it belongs according to Ji et al. (2019). The different PL states in each row are placed in ascending left-to-right order according to the likelihood of rejecting the null hypothesis when comparing their probability of occurrence between the groups, with the probability of the null hypotheses written above each respective PL state. Values in black represent an inability to reject the null hypothesis (*p* > 0.05), values in red represent a rejection of the null hypothesis with 95% confidence (*p* < 0.05), and values in green a rejection of the null hypothesis with 95% confidence that survives a conservative Bonferroni correction for the corresponding partitioning model (*p* < 0.05/*k*). The PL states that are referred to as the global mode of each partitioning model, in which all cortical parcels are approximately at the same phase, are plotted in this figure without any spheres and marked by a ^G^ after the value written above them. These states have the highest probability of occurrence in their respective partitioning model (see Supplementary Figure S1).

In line with previous applications of LEiDA (Alonso Martínez et al., 2020; Cabral et al., 2017; Farinha et al., 2022; Lord et al., 2019; Vohryzek et al., 2020, 2022), our analysis revealed a repertoire of phase-locking (PL) states in the resting-state fMRI signals that reoccurred over time and across participants. These PL states, obtained by clustering the instantaneous leading eigenvectors into a different number of clusters in each partitioning model (ranging from *k* = 2 to *k* = 20), can be characterized as dividing all cortical parcels to two communities according to their instantaneous phases. The smaller community is considered to be in anti-phase with the rest of the brain, and thus only the cortical parcels that belong to that community are rendered in the representation of the PL states (Figure 1B), with each rendered parcel colored according to the resting-state network to which it was exclusively assigned to in Ji et al. (2019), as detailed in Figure 1A. Each row shows the PL states obtained by increasing the number of clusters from 2 to 20, representing a separate partitioning model. Within each row, PL states are sorted according to the likelihood of occurring differently (i.e., rejecting the null hypothesis) when comparing their probability of occurrence between meditators and controls, with this likelihood increasing from left to right. Aligning with previous applications of LEiDA, each partitioning model contains one PL state referred to as the global mode, in which all parcels are relatively aligned in phase. These PL states are rendered in Figure 1B without the representation of any cortical parcels, as none are in anti-phase with the rest of the brain. These PL states have the highest probability of occurrence in their respective partitioning model (see Supplementary Figure S1), aligning the current study with other applications of the method. Having used a purely cortical and finer grained parcellation scheme, the patterns found here closely overlap with those obtained in fMRI data from the Human Connectome Project (Hancock et al., 2022; Vohryzek et al., 2020).

### Long-term meditation alters FC dynamics

**Figure 2:**
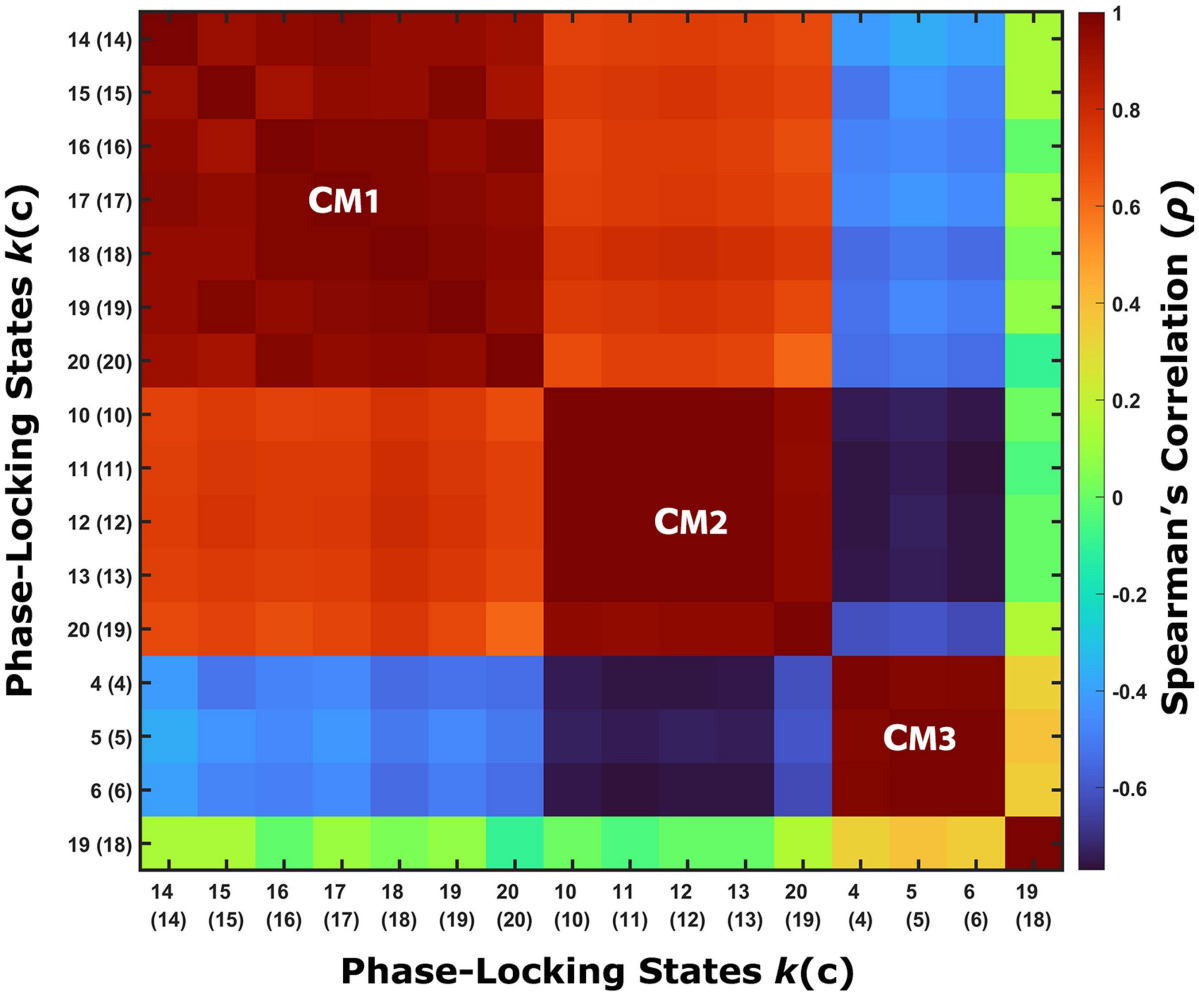
Phase-locking states that exhibit significant between-groups differences are similar between partitioning models. The correlations between the centroids of the phase-locking (PL) states that exhibit a significant between-groups difference in their probability of occurrence suggest dividing these PL states into three groups, referred to as coupling modes (CM). Each CM consists of highly correlated PL states from different partitioning models that share the same relationship between the groups (i.e., whether meditators spend more or less time in these PL states relative to the control group). PL states that involve posterior sensory areas compose CM1, while PL states that only involve a small number of specific cortical parcels associated with the secondary visual network belong to CM2, and PL states that involve higher cognitive areas are grouped into CM3. An additional PL state was found to exhibit a significant difference between the groups, but it was not correlated to any of the other groups, and therefore considered a partitioning-specific pattern (see Supplementary Figure S3).

**Figure 3:**
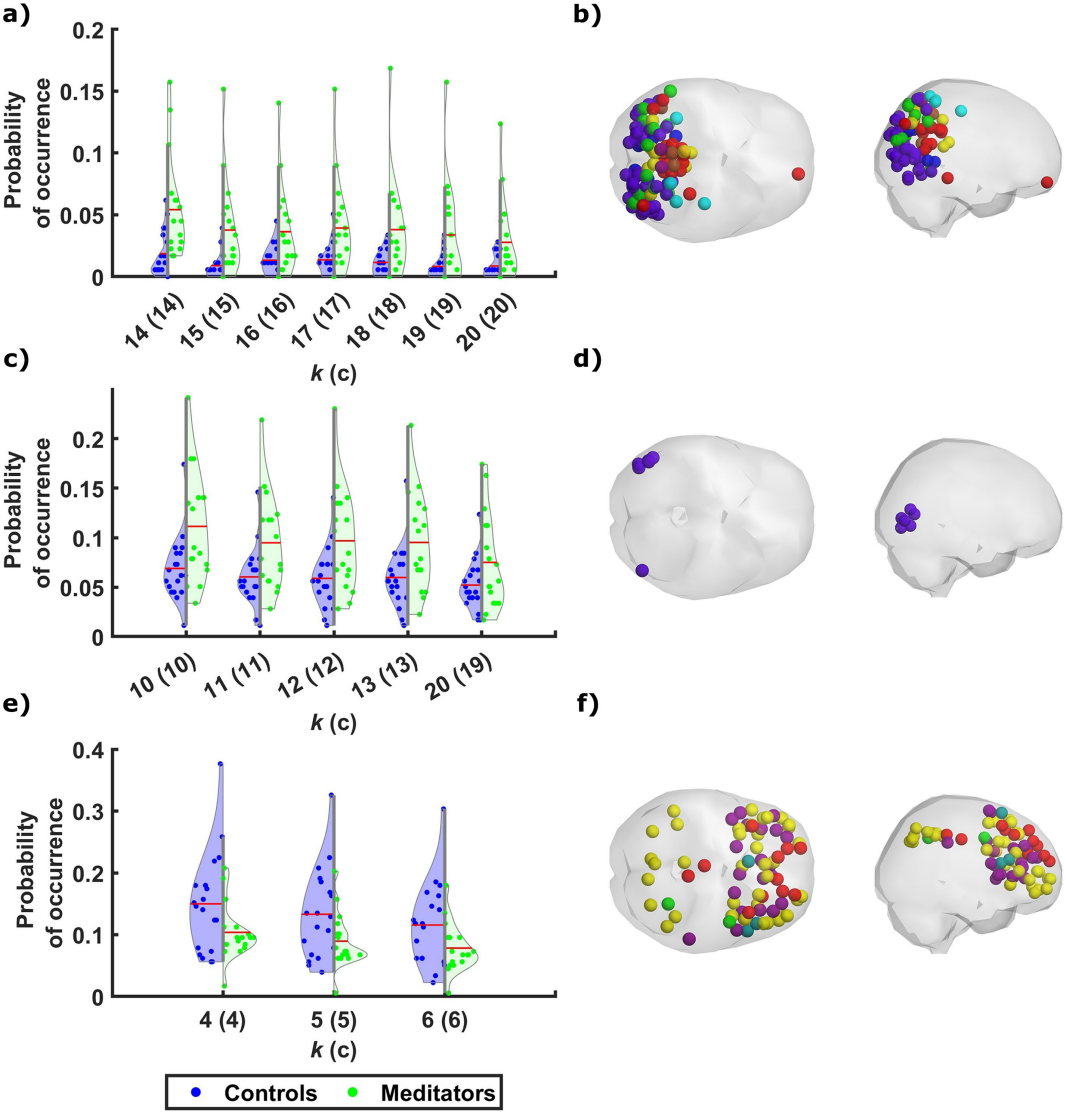
Meditators differ from controls in the probability of occurrence of phase-locking states of discernible neural networks. Across the different partitioning models, phase-locking (PL) states that exhibit a significant difference in probability of occurrence between the two groups fall into one of three patterns, along with a single state that is characterized by a separate pattern. The probability of occurrence of the individual PL states in CM1 **(A)** and CM2 **(C)** are significantly higher among meditators relative to controls, while the control participants spent more time on average in the PL states that belong to CM3 **(E)**. The mean probability of occurrence of each PL state among the participants of each group is marked by a red line. The cortical parcels involved in CM1, CM2, and CM3, are rendered (in **B**, **D**, and **F**, respectively) as spheres around their centers of gravity in axial and sagittal glass brains (middle and right, respectively), and colored according to their network affiliation as defined by Ji et al. (2019). The distribution of probabilities of occurrence among the two groups in another PL state that was not associated with any CM is shown in Supplementary Figure S3, along with the rendering of the relevant cortical parcels.

The probability of occurrence of each of the phase-locking (PL) states (Figure 1B) was compared between the long-term meditators (MM) and the control (C) participants. The clusters returning the most significant differences between groups are marked by highlighting the resulting *p*-values above the corresponding brain network images in either red or green, corresponding to *p* < 0.05 and corrected *p* < 0.05/*k*, respectively. Since the clusters are not independent for different *k* partitions, we grouped the clusters that showed significant differences into different coupling modes (CM), according to the correlations between them (Figure 2; see Supplementary Figure S2 for a visual comparison of the centroids of the individual PL states). Since the scores and probabilities are not normally distributed, Spearman’s rank correlation coefficient *ρ* was used in the entire study. The first coupling mode consists of PL states that were detected more often in meditators than in control participants (Table 2 CM 1; Figure 3A). These PL states mostly include regions that belong to primary and secondary visual networks, alongside several dorsal brain regions assigned to the dorsal attention network, DMN, and FPN (Figure 3B). The PL states in the second CM were also detected more often in meditators compared to controls (Table 2, CM 2; Figure 3C), highlighting a small group of regions from the secondary visual network (Figure 3D).

**Table 2:** Recurring PL patterns with significant between-groups differences in probability of occurrence.

Coupling Mode	*k*	Cluster	*p*-value	Probability of occurrence			Hedge’s *g*
				Controls		Meditators	
Posterior sensory areas (CM 1)	14	14	4.3E-04**	0.019 (0.0042)	<	0.054 (0.0102)	1.118
	15	15	6.1E-04**	0.009 (0.0026)	<	0.038 (0.0093)	1.041
	16	16	3.6E-03*	0.013 (0.0028)	<	0.036 (0.0087)	0.879
	17	17	2.2E-03**	0.014 (0.0030)	<	0.039 (0.0092)	0.931
	18	18	1.4E-03**	0.012 (0.0023)	<	0.038 (0.0099)	0.914
	19	19	4.1E-03*	0.008 (0.0023)	<	0.034 (0.0099)	0.879
	20	20	8.6E-03*	0.009 (0.0022)	<	0.028 (0.0081)	0.797
Visual areas (CM 2)	10	10	4.8E-03**	0.069 (0.0077)	<	0.111 (0.0135)	0.936
	11	11	7.6E-03*	0.060 (0.0068)	<	0.095 (0.0119)	0.866
	12	12	6.0E-03*	0.059 (0.0070)	<	0.097 (0.0127)	0.898
	13	13	8.9E-03*	0.060 (0.0073)	<	0.095 (0.0121)	0.857
	20	19	4.6E-02*	0.052 (0.0059)	<	0.075 (0.0117)	0.605
High cognitive areas (CM 3)	4	4	2.3E-02*	0.150 (0.0188)	>	0.104 (0.0110)	0.687
	5	5	2.1E-02*	0.133 (0.0168)	>	0.090 (0.0108)	0.710
	6	6	2.8E-02*	0.116 (0.0156)	>	0.078 (0.0096)	0.660
Partition specific pattern	19	18	3.4E-02*	0.026 (0.0086)	>	0.009 (0.0020)	0.579

Results of permutation-based non-parametric two sample hypothesis tests, pointing to recurring PL patterns that differ between the groups in terms of probability of occurrence. * - significant at *p* < 0.05; **significant at *p* < 0.05/k. Probabilities of occurrence are presented as mean across the participants of each group, followed by the standard error mean in parenthesis.

In contrast, the last CM of PL states was detected more often in control participants compared to meditators (Table 2, CM 3; Figure 3E). While the PL states in this CM did not survive the correction for multiple comparisons, they are quite consistent across partition models, involving coordination between latero-frontal and parietal components of the FPN and specific regions from the cingulo-opercular and default networks (Figure 3F). An additional pattern was found to differ between groups when clustering into *k* = 19 clusters (Table 2, Partition-specific pattern; Supplementary Figure S3), implicating brain areas distributed among ventral, dorsal, frontal, and parietal areas. However, given that comparing a higher number of states increases the chances of false positives and the difference did not survive correction, we did not consider this result in the subsequent analysis.

While the probability of occurrence of the global mode in all partitioning models did not significantly differ between the groups, meditators exhibited a slight preference of these PL states regardless of *k* (Supplementary Figure S4).

### Additional factors influencing FC dynamics

**Figure 4:**
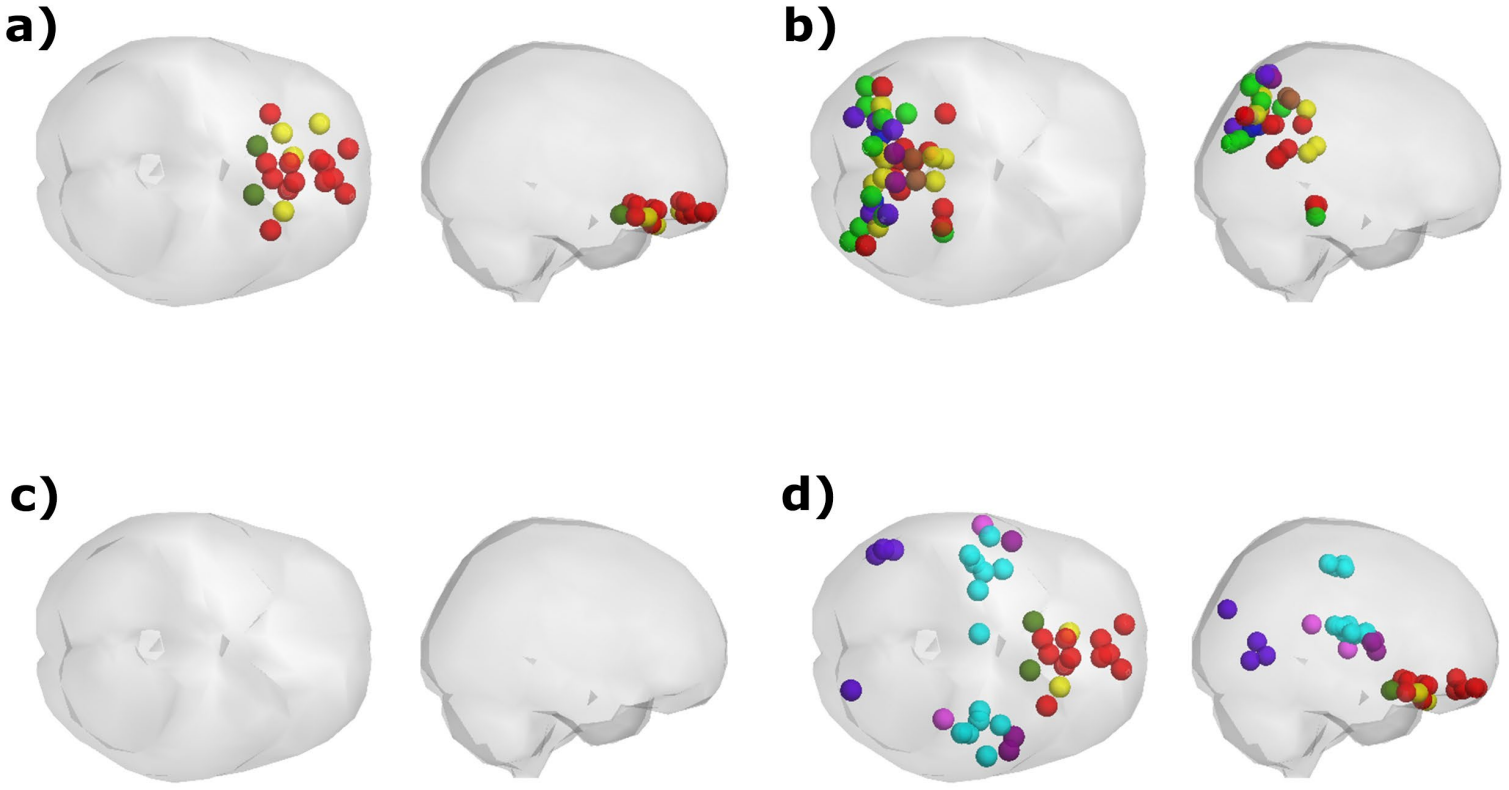
Visualization of the PL states that exhibit a significant correlation between their probability of occurrence and participants’ age and scores in Hood’s Mysticism Scale. **(A)** Age was positively correlated with the probability of occurrence of a PL pattern involving synchronization between orbitofrontal regions, regardless of meditation expertise. **(B)** Scores in the Unifying quality of Hood’s Mysticism Scale were positively correlated with the proportion of time spent in a PL state that involves posterior regions that are associated with various networks, although this correlation was found only in the *k* = 14 partitioning model. **(C)** Among meditators, age was negatively correlated with the probability of occurrence for the global mode, in which all cortical regions are in a similar phase. **(D)** meditators’ scores in the Noetic quality of the mysticism scale were found to be negatively correlated with the probability of occurrence for a PL pattern that was identified only by the *k* = 20 partitioning model.

The probabilities of occurrence of certain PL patterns were found to be correlated with either age or scores in the Hood’s Mysticism Scale (Table 3), but not with meditation expertise (among meditators). For the groups’ comparison in the Hood’s Mysticism Scale scores see Supplementary Table S1. Among all participants, irrespective of meditative experience or partitioning model, synchronization between certain parts of the anterior cingulate cortex (ACC), medial prefrontal cortex, and orbital or polar frontal cortex, was consistently detected more often in older participants (Table 3, Whole Sample; Figure 4A). The probability of occurrence of the global mode was negatively correlated with age among meditators (Supplementary Table S2), while a trend for positive correlations was found between age and probability of occurrence of the global state among the control participants in all partitioning models. However, these correlations achieved significance only in a subset of the partitioning models for the meditators group (Table 3, Meditators; Figure 4C), but not in any partitioning model for the control group. Moreover, no significant correlations were found for the control group between the probability of occurrence of any PL state, in any partitioning model, and their scores in any of the subscales of Hood’s Mysticism Scale.

**Table 3:** PL patterns that exhibit significant correlations between their respective probability of occurrence and participants’ age, scores in Hood’s Mysticism scale, or length of meditation practice.

Group	Measure	*k*	Cluster	Figure 4 panel	Direction	Spearman’s *ρ*	Holm-Bonferroni corrected *p*-value
Whole sample	Age	6	5	A	Positive	0.561	2.2E-02
	Age	7	5	A	Positive	0.567	2.2E-02
	Age	8	6	A	Positive	0.555	3.5E-02
	Age	10	6	A	Positive	0.578	2.2E-02
	Age	11	4	A	Positive	0.587	1.8E-02
	Age	12	5	A	Positive	0.596	1.5E-02
	Age	13	9	A	Positive	0.589	2.1E-02
	Age	14	10	A	Positive	0.59	2.1E-02
	Age	15	8	A	Positive	0.569	4.4E-02
	Age	16	7	A	Positive	0.604	1.5E-02
	Age	17	7	A	Positive	0.598	2.0E-02
	Age	18	10	A	Positive	0.589	2.9E-02
	Age	19	8	A	Positive	0.603	1.9E-02
	Unifying quality	14	14	B	Positive	0.669	1.6E-03
Meditators	Age	6	4	C	Negative	−0.793	1.0E-02
	Age	7	3	C	Negative	−0.817	5.3E-03
	Age	8	3	C	Negative	−0.782	2.0E-02
	Age	11	2	C	Negative	−0.788	2.3E-02
	Age	12	4	C	Negative	−0.78	3.1E-02
	Age	13	1	C	Negative	−0.785	2.9E-02
	Age	18	2	C	Negative	−0.78	4.9E-02
	Noetic quality	20	1	D	Negative	−0.822	2.2E-02

Spearman’s correlations were calculated between participants’ age and scores in Hood’s Mysticism scale. For the meditators, length of mediation practice was also tested for correlation. Visual representation of the relevant PL patterns is found in the respective panel in Figure 4.

Two of the PL patterns exhibited significant correlations between their respective probability of occurrence and scores of the Mysticism scale. Both patterns encompass regions that are both spatially distributed in the brain and are associated with a variety of documented functional networks. Across the entire sample, scores in the Unifying quality aspect of the scale were found to be positively correlated with the probability of occurrence of a PL pattern composed of posterior regions that mostly belong to the visual, frontoparietal, dorsal attention, and default networks (Table 3, Whole Sample; Figure 4B). Meditators exhibited a negative correlation between their scores for ‘Noetic Quality’ and the probability of occurrence for a state in which synchronization occurs between orbitofrontal regions from the DMN, alongside regions from the somatomotor and secondary visual networks (Table 3, Meditators; Figure 4D).

## Discussion

The aim of this study was to examine the dynamics of functional connectivity (FC) that characterize long-term MM practitioners. This was achieved using the LEiDA approach on resting-state fMRI scans of long-term meditators and age-matched controls. Similar to other studies that use the LEiDA toolset, our analysis revealed a wide repertoire of transient PL states that reflect the transient cooperation between nodes forming different functional networks over time (Martínez et al., 2020; Cabral et al., 2017; Farinha et al., 2022; Lord et al., 2019; Vohryzek et al., 2020, 2022). By comparing the probability of occurrence of these phase-locking (PL) states between the groups, we found differences suggesting that long-term MM practice may impact the dynamics of the interactions between different brain regions even during rest. Examining the centroids of the states that exhibit between-groups differences in their probability of occurrence revealed several patterns that were consistent across partitions with different numbers of clusters. These PL patterns were categorized in three distinct coupling modes (CMs) according to their spatial similarities.

While the analysis performed here was exploratory in nature, the results were validated with rigorous statistical testing and conservative corrections for multiple testing, ensuring higher chances of being true positives. Unlike other studies of dynamic FC, we opted not to focus on a single partitioning model, since similar patterns that maximize the differences between groups appeared for different models. This could possibly stem from the differences in granularity between the models, which might result in the obfuscation of between-groups differences in certain models, being too coarse or too fine, to identify the relevant connectivity patterns.

We demonstrate here that compared to controls, meditators exhibit increased occupancy in a CM that involves coordination between parcels of several networks, mainly the visual and dorsal attention networks, with a smaller contribution of parcels from the FPN, DMN, and posterior multimodal network (Figures 3A,B). All the parcels involved in these PL states are situated posteriorly in the brain, i.e., within the occipital, parietal, and posterior temporal lobes. This pattern resembles other neuroimaging meditation studies reporting enhanced FC within posterior brain regions, including the dorsal attention network during meditation (Froeliger et al., 2012), and sensory regions following short-term meditation training (Kilpatrick et al., 2011). The increased occupancy of this pattern among meditators compared to controls could signify a greater capacity for disengagement from self-related thoughts (Northoff et al., 2006; Qin and Northoff, 2011), and a stronger inclination to shift attention toward attentional, affective, and sensory processes, a notion that is in line with those of the original study (Berkovich-Ohana et al., 2016a). The development of such a capacity has been attributed to long-term MM practice (Tang et al., 2015), both as a state effect (Farb et al., 2007; Pagnoni, 2012) and as a trait reflected in resting-state activity (Escrichs et al., 2019; Garrison et al., 2015; Yellin et al., 2015). A similar PL pattern was previously found to be more frequently occupied by participants with relatively lower depressive symptoms following a relationship breakup (Alonso Martínez et al., 2020). This similarity of findings supports studies that emphasize the beneficial impacts of MM in treating depression (Chi et al., 2018; Reangsing et al., 2021) and enhancing emotion regulation (Davidson et al., 2003; Farb et al., 2012).

Compared to controls, meditators also spent a greater proportion of time in another CM (Figures 3C,D) that consists of several lateral occipital and posterior temporal regions, affiliated to the secondary visual network. Using the terminology defined by Glasser et al. (2016), the regions involved in these states consist mainly of the right middle temporal area, right lateral occipital 2 and bilateral area V4t. The regions included in this PL pattern are extra-striate, high-order visual association areas, involved with visual motion perception. Previous analyses of the functional data of the participants involved in this study have shown enhanced activation (Berkovich-Ohana et al., 2016a) and connectivity (Berkovich-Ohana et al., 2016b) in some of the regions included in this pattern during a visual recognition memory task among meditators compared with controls.

The occupancy in a third CM was found to be significantly greater amongst controls compared to meditators, though the significance of this effect did not survive multiple comparison correction (Figures 3E,F). Most of the parcels involved belong to the FPN or cingulo-opercular network, alongside a few parcels from the DMN. These regions are associated with high-order cognitive control and executive functions, as well as self-related processes. The decreased occupancy of meditators in these PL states resembles the decreased probability of occurrence found in similar states following the injection of psilocybin (Lord et al., 2019; Olsen et al., 2022).

Interestingly, we did not detect significant between-group differences in probability of occurrence in any of the PL states that exhibited a greater involvement of the DMN as a whole, which has been shown to function differently among long-term meditators (Tang et al., 2015). This seems to counter a previous study that has shown a DMN microstate with higher duration and probability of occurrence among experienced practitioners of Raja Yoga when compared to non-practitioners (Panda et al., 2016). This suggests that different meditative practices lead to distinct effects on the brain. The effects of long-term mindfulness practice are likely more complex, as a different study has detected reduction in the probability of occurrence of DMN-involving state among meditators during meditation, compared to during rest (Dagnino et al., 2024). Nevertheless, our analysis did find differences in the involvement of sub-areas of the DMN in the PL states that distinguish meditators from control participants. Meditators spent more time in states that mostly encompass posterior regions of the DMN, including the ventral and dorsal PCC (BA23 and BA31, respectively) and the medial precuneus (BA7), which are considered to support bottom-up processes, self-generated tasks, autonomic arousal, and awareness. The angular gyrus (PGs and PGi) was also detected by this analysis, known to function as a cross-modal hub, allowing internal and perceptual sources of information to access conceptual representations about events or items in their spatiotemporal context (Andrews-Hanna et al., 2014). In contrast, controls spent more time in states that mostly involved prefrontal nodes of the DMN, which are considered to also support top-down processes related to various executive functions (Miller, 2007). These include parts of the dorsal ACC (BA32), which is associated with cognitive control (Shenhav et al., 2016) and reward-based decision making (Bush et al., 2002); dorsomedial portions of the prefrontal cortex (medial parts of BA8 and BA9), which have been associated with predicting the likely outcomes of actions (Alexander and Brown, 2011); and dorsolateral portions of the prefrontal cortex (lateral portions of BA8), considered relevant to working memory (Barbey et al., 2013) and other cognitive demands (Duncan and Owen, 2000).

Our results also show that the spontaneous occurrence of FC patterns is affected, at least to some extent, by processes that accompany natural aging. Synchronization between a set of orbitofrontal regions was detected more often among older participants from both groups, while the occurrence of the global mode was found to decrease with age among meditators. The global mode, shown here to decrease with age among meditators, has been previously associated with higher cognitive performance (Cabral et al., 2017), and found to be increased after psilocybin injection (Lord et al., 2019; Olsen et al., 2022). In contrast, schizophrenia patients (Farinha et al., 2022) were observed to spend less time in this mode. While the correlations detected in the current study between age and time spent in the global mode are significant in several partitioning models, their interpretation requires further investigation of the correspondence between the global mode detection and specific neural functions.

Correlations with scores of the Mysticism scale were found with two PL patterns, each specific to a single partitioning model and encompassing regions that are distributed both spatially and in terms of network association. The positive correlation between participants’ scores in the Unifying quality index and the probability of occurrence of a PL pattern that involves posterior, sensory processing-related regions, might relate to the detection of the same pattern as occurring more frequently among meditators, possibly reflecting heightened engagement in sensory processes among meditators. However, caution is required here, as both this pattern and its correlation are specific to a single partitioning model and could therefore be insufficiently robust. Nevertheless, these results might invite further research to understand how, and if, to interpret them.

Overall, the results of the current study support the potential of MM to impact brain function by altering the dynamics of functional connections between the different brain regions, even outside of meditation (i.e., as a long-term trait effect), suggesting that MM may increase the stability of neural states that support embodied momentary bottom-up cognition, shifting away from top-down reflective self-related processing.

### Limitations and future directions

The current study bears several limitations. First, as with any cross-sectional study, it is difficult to disentangle the effects of meditation and those of other lifestyle parameters. Second, the use of the extensive Glasser atlas in the present study meant that each observation (i.e., each time point for each participant) contains many features, which renders the study underpowered and warrants a larger participant pool than available. This also led to the decision to limit the focus of the analyses on the cortical parcels defined by Glasser et al. (2016), without including the sub-cortical expansion performed by Ji et al. (2019). Future studies could produce more robust results by using a larger sample size and should implement a longitudinal design to better distinguish between the effects of mindfulness meditation practice from those of other life experiences. In addition, examining FC dynamics during the practice of various forms of meditation could provide more detailed information regarding the effects of these practices. Another possibility would be to measure the performance of experienced mindfulness practitioners in certain tasks, with the aim of evaluating any potential link between the impact of this practice on cognitive functions. Lastly, future studies could apply temporal filtering to the data and evaluate the effects of long-term mindfulness practice on specific frequency components of the fMRI signal.

## Data Availability

Publicly available datasets were analyzed in this study. This data can be found at: https://drive.google.com/drive/folders/13BiUSHmigMIvknIoMYwx0pbVFNPugTil?usp=share_link.

## Footnotes

^1^ https://github.com/xiangruili/dicm2nii

^2^ https://www.mathworks.com/matlabcentral/fileexchange/28303-bonferroni-holm-correction-for-multiple-comparisons
