# Supplementary material for: Long-term mindfulness meditation increases occurrence of sensory and attention brain states
Source: Front Hum Neurosci. 2025 Jan 6;18:1482353. doi: 10.3389/fnhum.2024.1482353 (PMC11743700; doi:10.3389/fnhum.2024.1482353)
Supplement: Supplementary file 1 [file Data_Sheet_1.docx]

**Supplementary Materials**

**Supplementary Figures**


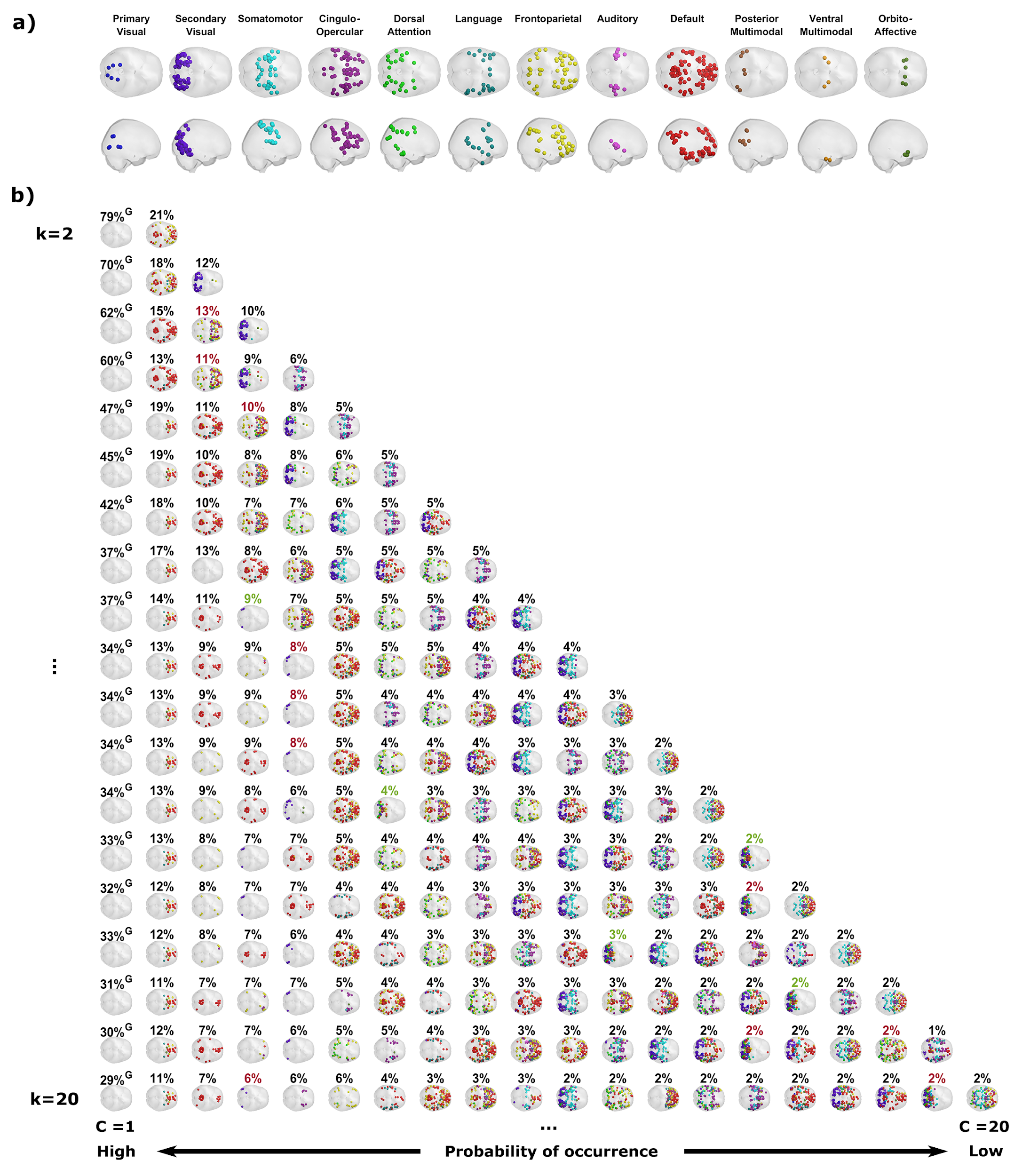


**Supplementary Figure S1. Recurring functional connectivity patterns detected by partitions into a varying number of states.** (a) The network affiliation of each brain parcel according to Ji et al. (2019). (b) The clustering procedure reveals the PL states for each partition model, plotted as separate rows. The phase-locking (PL) states are represented as a set of spheres at the center of gravity of the cortical parcels that are in anti-phase with respect to the rest of the brain, with their respective mean probability of occurrence across all participants presented above them. Each sphere is colored to match the established resting state network to which it belongs according to Ji et al. (2019). The different PL states in each row are placed in descending left-to-right order according to their probability of occurrence, starting with the global mode (marked by ^G^), which is the dominant state for the largest portion of time among all participants. States that exhibit a significant difference in their probability of occurrence between meditators and control participants have their probability of occurrence highlighted in either red or green, corresponding to p<0.05 and corrected p<0.05/*k*, respectively.


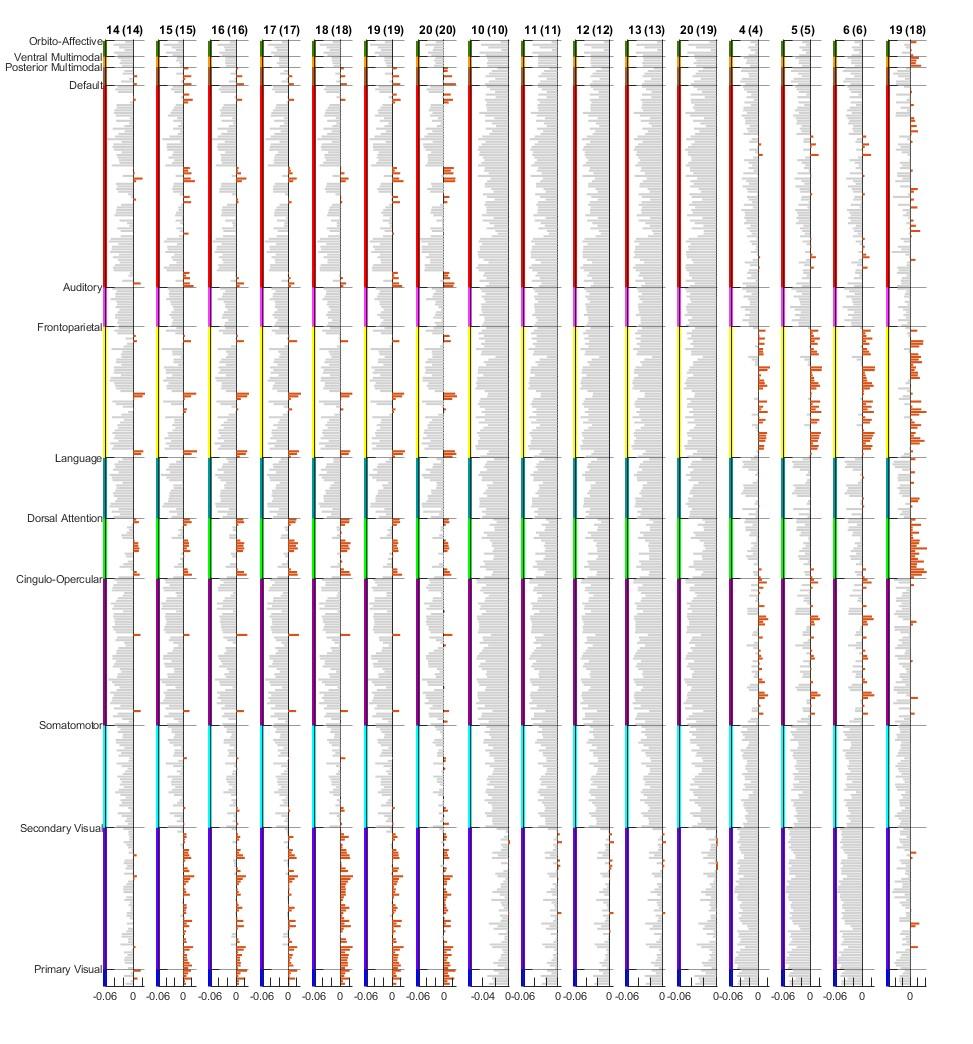


CM1

CM2

CM3

**Supplementary Figure S2. Recurring functional connectivity patterns detected by partitions into a varying number of states.** The phase-locking (PL) pattern of each state that exhibits significant differences between the groups in terms of probability of occurrence are shown here in the form of their respective centroid, which were produced by the *k*-means partitioning procedure, with positive values representing the cortical parcels involved in each PL state. These centroids are labeled according to the partitioning model in which they were found (represented by the number of clusters, *k*), followed by the number of the cluster c in parentheses (according to Figure 1b). In order to show the similarity between the centroids of each coupling mode (CM), they are grouped according to the CM to which they belong.


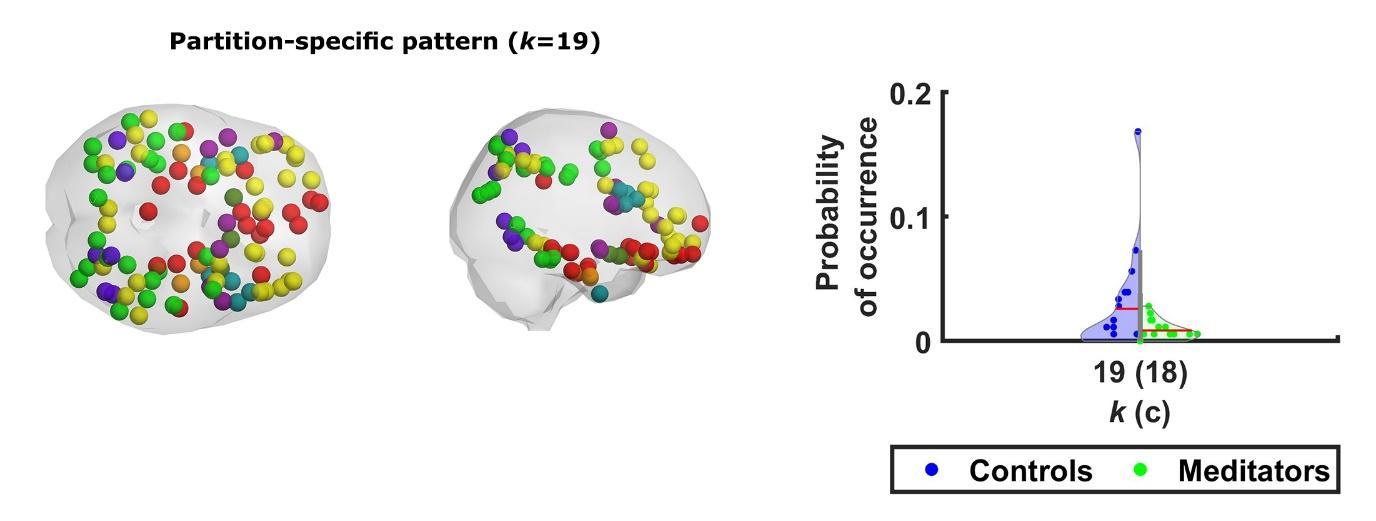


**Supplementary Figure S3. Relative to controls, meditators spent a smaller portion of time in a partition-specific connectivity pattern.** The cortical parcels involved in this PL state are rendered as spheres around their centers of gravity in axial and sagittal glass brains (left and middle, respectively), and colored according to their network affiliation as defined by Ji et al. (2019). While the probability of occurrence of the PL state was smaller among meditators than among controls (*p*=0.0034; did not survive the correction for multiple comparisons), no other PL state with a similar centroid exhibited between-groups differences.


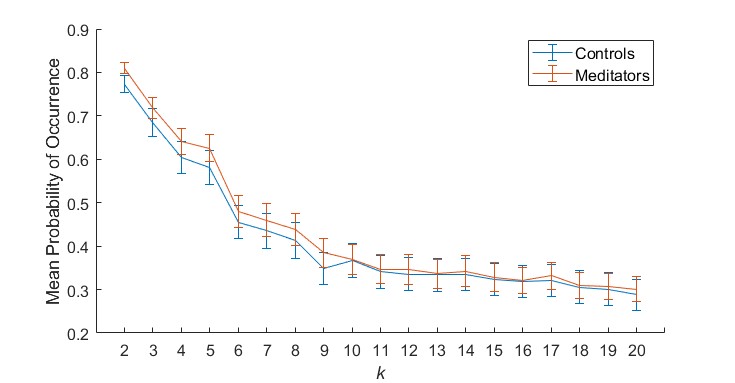


**Supplementary Figure S4. The probability of occurrence of the global mode states of the different partitioning models were slightly higher among meditators than controls.** Although the differences between the groups in the probability of occurrence of the global mode states were not significant, a visual inspection of these probabilities of occurrence reveals a weak preference of these states among meditators compared to controls.

**Supplementary Tables**

**Supplementary Table S1. Meditators score higher than controls in most subscales of Hood’s Mysticism Scale**

| **Quality** | **Controls (C)** | | **Meditators (MM)** | | **Bonferroni Corrected *p*** |
| --- | --- | --- | --- | --- | --- |
|  | **Mean** | **SEM** | **Mean** | **SEM** |  |
| **Ego** | 2.93 | 0.257 | 4.25 | 0.180 | 0.008* |
| **Unifying** | 2.54 | 0.300 | 4.17 | 0.222 | 0.007* |
| **Inner Subjective** | 2.62 | 0.303 | 3.77 | 0.247 | 0.109 |
| **Temporal/Spatial** | 3.39 | 0.311 | 4.52 | 0.199 | 0.059 |
| **Noetic** | 3.21 | 0.184 | 4.44 | 0.166 | 0.001* |
| **Ineffability** | 3.68 | 0.197 | 4.59 | 0.180 | 0.017* |
| **Positive Affect** | 4.05 | 0.201 | 4.80 | 0.092 | 0.020* |
| **Religious** | 2.89 | 0.318 | 4.13 | 0.211 | 0.093 |
| **Mean** | 3.18 | 0.189 | 4.33 | 0.116 | 0.001* |

The scores of participants from the different groups for each subscale of Hood’s Mysticism Scale were compared using Wilcoxon rank sum test. Following Bonferroni correction for multiple comparisons, meditators were found to score significantly higher than controls in the Ego, Unifying, Noetic, Ineffability, and Positive Affect qualities, as well as in the overall score (Mean). No significant differences were found between the groups in the Inner Subjective, Temporal/Spatial, and Religious qualities. *p*-values marked by an * indicate significant differences that survive a Bonferroni correction for multiple comparisons.

**Supplementary Table S2. Participants’ age was found to be significantly correlated with the probability of occurrence of the global mode state among meditators, but not among controls**

| *k* | Controls (C) | | Meditators (MM) | |
| --- | --- | --- | --- | --- |
|  | Spearman's *ρ* | Holm-Bonferroni Corrected *p* | Spearman's *ρ* | Holm-Bonferroni Corrected *p* |
| 2 | 0.535 | 0.368 | -0.230 | 1.000 |
| 3 | 0.288 | 1.000 | -0.422 | 1.000 |
| 4 | 0.400 | 1.000 | -0.431 | 1.000 |
| 5 | 0.396 | 1.000 | -0.441 | 1.000 |
| 6 | 0.179 | 1.000 | -0.793 | 0.010* |
| 7 | 0.251 | 1.000 | -0.817 | 0.005* |
| 8 | 0.252 | 1.000 | -0.782 | 0.020* |
| 9 | 0.330 | 1.000 | -0.659 | 0.430 |
| 10 | 0.285 | 1.000 | -0.753 | 0.058 |

Expanding on Table 3, the results of Spearman’s correlations between participants’ age and their probability of occurrence of the global mode states in various partitioning models are presented here for each group separately. Although not all were significant, the correlations between age and the probability of occurrence of the global mode state were positive among controls and negative among meditators. Significant correlations with age (marked by an *) were found for the global mode state of some of the partitioning models among meditators, but not among controls.

**Supplementary Table S2 - Continued.**

| *k* | Controls (C) | | Meditators (MM) | |
| --- | --- | --- | --- | --- |
|  | Spearman's *ρ* | Holm-Bonferroni Corrected *p* | Spearman's *ρ* | Holm-Bonferroni Corrected *p* |
| 11 | 0.395 | 1.000 | -0.788 | 0.023* |
| 12 | 0.334 | 1.000 | -0.780 | 0.031* |
| 13 | 0.352 | 1.000 | -0.785 | 0.029* |
| 14 | 0.359 | 1.000 | -0.744 | 0.105 |
| 15 | 0.399 | 1.000 | -0.770 | 0.053 |
| 16 | 0.393 | 1.000 | -0.746 | 0.111 |
| 17 | 0.386 | 1.000 | -0.773 | 0.056 |
| 18 | 0.428 | 1.000 | -0.780 | 0.049* |
| 19 | 0.404 | 1.000 | -0.758 | 0.097 |
| 20 | 0.476 | 1.000 | -0.711 | 0.332 |

**Supplementary Methods**

Results included in this manuscript come from preprocessing performed using *fMRIPrep* 20.2.1 (Esteban, Markiewicz, et al. (2018); Esteban, Blair, et al. (2018); RRID:SCR_016216), which is based on *Nipype* 1.5.1 (Gorgolewski et al. (2011); Gorgolewski et al. (2018); RRID:SCR_002502).

**Anatomical data preprocessing**

A total of 1 T1-weighted (T1w) images were found within the input BIDS dataset.The T1-weighted (T1w) image was corrected for intensity non-uniformity (INU) with N4BiasFieldCorrection (Tustison et al. 2010), distributed with ANTs 2.3.3 (Avants et al. 2008, RRID:SCR_004757), and used as T1w-reference throughout the workflow. The T1w-reference was then skull-stripped with a *Nipype* implementation of the antsBrainExtraction.sh workflow (from ANTs), using OASIS30ANTs as target template. Brain tissue segmentation of cerebrospinal fluid (CSF), white-matter (WM) and gray-matter (GM) was performed on the brain-extracted T1w using fast (FSL 5.0.9, RRID:SCR_002823, Zhang, Brady, and Smith 2001). Brain surfaces were reconstructed using recon-all (FreeSurfer 6.0.1, RRID:SCR_001847, Dale, Fischl, and Sereno 1999), and the brain mask estimated previously was refined with a custom variation of the method to reconcile ANTs-derived and FreeSurfer-derived segmentations of the cortical gray-matter of Mindboggle (RRID:SCR_002438, Klein et al. 2017). Volume-based spatial normalization to one standard space (MNI152NLin2009cAsym) was performed through nonlinear registration with antsRegistration (ANTs 2.3.3), using brain-extracted versions of both T1w reference and the T1w template. The following template was selected for spatial normalization: *ICBM 152 Nonlinear Asymmetrical template version 2009c* [Fonov et al. (2009), RRID:SCR_008796; TemplateFlow ID: MNI152NLin2009cAsym],

**Functional data preprocessing**

For each of the 1 BOLD runs found per subject (across all tasks and sessions), the following preprocessing was performed. First, a reference volume and its skull-stripped version were generated using a custom methodology of *fMRIPrep*. Susceptibility distortion correction (SDC) was omitted. The BOLD reference was then co-registered to the T1w reference using bbregister (FreeSurfer) which implements boundary-based registration (Greve and Fischl 2009). Co-registration was configured with six degrees of freedom. Head-motion parameters with respect to the BOLD reference (transformation matrices, and six corresponding rotation and translation parameters) are estimated before any spatiotemporal filtering using mcflirt (FSL 5.0.9, Jenkinson et al. 2002). BOLD runs were slice-time corrected using 3dTshift from AFNI 20160207 (Cox and Hyde 1997, RRID:SCR_005927). The BOLD time-series (including slice-timing correction when applied) were resampled onto their original, native space by applying the transforms to correct for head-motion. These resampled BOLD time-series will be referred to as *preprocessed BOLD in original space*, or just *preprocessed BOLD*. The BOLD time-series were resampled into standard space, generating a *preprocessed BOLD run in MNI152NLin2009cAsym space*. First, a reference volume and its skull-stripped version were generated using a custom methodology of *fMRIPrep*. Several confounding time-series were calculated based on the *preprocessed BOLD*: framewise displacement (FD), DVARS and three region-wise global signals. FD was computed using two formulations following Power (absolute sum of relative motions, Power et al. (2014)) and Jenkinson (relative root mean square displacement between affines, Jenkinson et al. (2002)). FD and DVARS are calculated for each functional run, both using their implementations in *Nipype* (following the definitions by Power et al. 2014). The three global signals are extracted within the CSF, the WM, and the whole-brain masks. Additionally, a set of physiological regressors were extracted to allow for component-based noise correction (*CompCor*, Behzadi et al. 2007). Principal components are estimated after high-pass filtering the *preprocessed BOLD* time-series (using a discrete cosine filter with 128s cut-off) for the two *CompCor* variants: temporal (tCompCor) and anatomical (aCompCor). tCompCor components are then calculated from the top 2% variable voxels within the brain mask. For aCompCor, three probabilistic masks (CSF, WM and combined CSF+WM) are generated in anatomical space. The implementation differs from that of Behzadi et al. in that instead of eroding the masks by 2 pixels on BOLD space, the aCompCor masks are subtracted a mask of pixels that likely contain a volume fraction of GM. This mask is obtained by dilating a GM mask extracted from the FreeSurfer’s *aseg* segmentation, and it ensures components are not extracted from voxels containing a minimal fraction of GM. Finally, these masks are resampled into BOLD space and binarized by thresholding at 0.99 (as in the original implementation). Components are also calculated separately within the WM and CSF masks. For each CompCor decomposition, the *k* components with the largest singular values are retained, such that the retained components’ time series are sufficient to explain 50 percent of variance across the nuisance mask (CSF, WM, combined, or temporal). The remaining components are dropped from consideration. The head-motion estimates calculated in the correction step were also placed within the corresponding confounds file. The confound time series derived from head motion estimates and global signals were expanded with the inclusion of temporal derivatives and quadratic terms for each (Satterthwaite et al. 2013). Frames that exceeded a threshold of 0.5 mm FD or 1.5 standardised DVARS were annotated as motion outliers. All resamplings can be performed with *a single interpolation step* by composing all the pertinent transformations (i.e. head-motion transform matrices, susceptibility distortion correction when available, and co-registrations to anatomical and output spaces). Gridded (volumetric) resamplings were performed using antsApplyTransforms (ANTs), configured with Lanczos interpolation to minimize the smoothing effects of other kernels (Lanczos 1964). Non-gridded (surface) resamplings were performed using mri_vol2surf (FreeSurfer).

Many internal operations of *fMRIPrep* use *Nilearn* 0.6.2 (Abraham et al. 2014, RRID:SCR_001362), mostly within the functional processing workflow. For more details of the pipeline, see [the section corresponding to workflows in *fMRIPrep*’s documentation](https://fmriprep.readthedocs.io/en/latest/workflows.html).

**Copyright Waiver**

The above boilerplate text was automatically generated by fMRIPrep with the express intention that users should copy and paste this text into their manuscripts *unchanged*. It is released under the [CC0](https://creativecommons.org/publicdomain/zero/1.0/) license.

**References**

Abraham, Alexandre, Fabian Pedregosa, Michael Eickenberg, Philippe Gervais, Andreas Mueller, Jean Kossaifi, Alexandre Gramfort, Bertrand Thirion, and Gael Varoquaux. 2014. “Machine Learning for Neuroimaging with Scikit-Learn.” *Frontiers in Neuroinformatics* 8. <https://doi.org/10.3389/fninf.2014.00014>.

Avants, B.B., C.L. Epstein, M. Grossman, and J.C. Gee. 2008. “Symmetric Diffeomorphic Image Registration with Cross-Correlation: Evaluating Automated Labeling of Elderly and Neurodegenerative Brain.” *Medical Image Analysis* 12 (1): 26–41. <https://doi.org/10.1016/j.media.2007.06.004>.

Behzadi, Yashar, Khaled Restom, Joy Liau, and Thomas T. Liu. 2007. “A Component Based Noise Correction Method (CompCor) for BOLD and Perfusion Based fMRI.” *NeuroImage* 37 (1): 90–101. <https://doi.org/10.1016/j.neuroimage.2007.04.042>.

Cox, Robert W., and James S. Hyde. 1997. “Software Tools for Analysis and Visualization of fMRI Data.” *NMR in Biomedicine* 10 (4-5): 171–78. [https://doi.org/10.1002/(SICI)1099-1492(199706/08)10:4/5<171::AID-NBM453>3.0.CO;2-L](https://doi.org/10.1002/(SICI)1099-1492(199706/08)10:4/5%3c171::AID-NBM453%3e3.0.CO;2-L).

Dale, Anders M., Bruce Fischl, and Martin I. Sereno. 1999. “Cortical Surface-Based Analysis: I. Segmentation and Surface Reconstruction.” *NeuroImage* 9 (2): 179–94. <https://doi.org/10.1006/nimg.1998.0395>.

Esteban, Oscar, Ross Blair, Christopher J. Markiewicz, Shoshana L. Berleant, Craig Moodie, Feilong Ma, Ayse Ilkay Isik, et al. 2018. “FMRIPrep.” *Software*. Zenodo. <https://doi.org/10.5281/zenodo.852659>.

Esteban, Oscar, Christopher Markiewicz, Ross W Blair, Craig Moodie, Ayse Ilkay Isik, Asier Erramuzpe Aliaga, James Kent, et al. 2018. “fMRIPrep: A Robust Preprocessing Pipeline for Functional MRI.” *Nature Methods*. <https://doi.org/10.1038/s41592-018-0235-4>.

Fonov, VS, AC Evans, RC McKinstry, CR Almli, and DL Collins. 2009. “Unbiased Nonlinear Average Age-Appropriate Brain Templates from Birth to Adulthood.” *NeuroImage* 47, Supplement 1: S102. <https://doi.org/10.1016/S1053-8119(09)70884-5>.

Gorgolewski, K., C. D. Burns, C. Madison, D. Clark, Y. O. Halchenko, M. L. Waskom, and S. Ghosh. 2011. “Nipype: A Flexible, Lightweight and Extensible Neuroimaging Data Processing Framework in Python.” *Frontiers in Neuroinformatics* 5: 13. <https://doi.org/10.3389/fninf.2011.00013>.

Gorgolewski, Krzysztof J., Oscar Esteban, Christopher J. Markiewicz, Erik Ziegler, David Gage Ellis, Michael Philipp Notter, Dorota Jarecka, et al. 2018. “Nipype.” *Software*. Zenodo. <https://doi.org/10.5281/zenodo.596855>.

Greve, Douglas N, and Bruce Fischl. 2009. “Accurate and Robust Brain Image Alignment Using Boundary-Based Registration.” *NeuroImage* 48 (1): 63–72. <https://doi.org/10.1016/j.neuroimage.2009.06.060>.

Jenkinson, Mark, Peter Bannister, Michael Brady, and Stephen Smith. 2002. “Improved Optimization for the Robust and Accurate Linear Registration and Motion Correction of Brain Images.” *NeuroImage* 17 (2): 825–41. <https://doi.org/10.1006/nimg.2002.1132>.

Klein, Arno, Satrajit S. Ghosh, Forrest S. Bao, Joachim Giard, Yrjö Häme, Eliezer Stavsky, Noah Lee, et al. 2017. “Mindboggling Morphometry of Human Brains.” *PLOS Computational Biology* 13 (2): e1005350. <https://doi.org/10.1371/journal.pcbi.1005350>.

Lanczos, C. 1964. “Evaluation of Noisy Data.” *Journal of the Society for Industrial and Applied Mathematics Series B Numerical Analysis* 1 (1): 76–85. <https://doi.org/10.1137/0701007>.

Power, Jonathan D., Anish Mitra, Timothy O. Laumann, Abraham Z. Snyder, Bradley L. Schlaggar, and Steven E. Petersen. 2014. “Methods to Detect, Characterize, and Remove Motion Artifact in Resting State fMRI.” *NeuroImage* 84 (Supplement C): 320–41. <https://doi.org/10.1016/j.neuroimage.2013.08.048>.

Satterthwaite, Theodore D., Mark A. Elliott, Raphael T. Gerraty, Kosha Ruparel, James Loughead, Monica E. Calkins, Simon B. Eickhoff, et al. 2013. “An improved framework for confound regression and filtering for control of motion artifact in the preprocessing of resting-state functional connectivity data.” *NeuroImage* 64 (1): 240–56. <https://doi.org/10.1016/j.neuroimage.2012.08.052>.

Tustison, N. J., B. B. Avants, P. A. Cook, Y. Zheng, A. Egan, P. A. Yushkevich, and J. C. Gee. 2010. “N4ITK: Improved N3 Bias Correction.” *IEEE Transactions on Medical Imaging* 29 (6): 1310–20. <https://doi.org/10.1109/TMI.2010.2046908>.

Zhang, Y., M. Brady, and S. Smith. 2001. “Segmentation of Brain MR Images Through a Hidden Markov Random Field Model and the Expectation-Maximization Algorithm.” *IEEE Transactions on Medical Imaging* 20 (1): 45–57. <https://doi.org/10.1109/42.906424>.
